# Galectin-3 in Inflammasome Activation and Primary Biliary Cholangitis Development

**DOI:** 10.3390/ijms21145097

**Published:** 2020-07-19

**Authors:** Aleksandar Arsenijevic, Bojana Stojanovic, Jelena Milovanovic, Dragana Arsenijevic, Nebojsa Arsenijevic, Marija Milovanovic

**Affiliations:** 1 Center for Molecular Medicine and Stem Cell Research, Faculty of Medical Sciences, University of Kragujevac, Kragujevac 34000, Serbia; aleksandar@medf.kg.ac.rs (A.A.); bojana.stojanovic04@gmail.com (B.S.); jelenamilovanovic205@gmail.com (J.M.); arne@medf.kg.ac.rs (N.A.); 2 Department of Pathophysiology, Faculty of Medical Sciences, University of Kragujevac, Kragujevac 34000, Serbia; 3 Department of Histology, Faculty of Medical Sciences, University of Kragujevac, Kragujevac 34000, Serbia; 4 Department of Pharmacy, Faculty of Medical Sciences, University of Kragujevac, Kragujevac 34000, Serbia; menki@hotmail.rs

**Keywords:** primary biliary cholangitis (PBC), galectin-3, NLRP3, inflammasome

## Abstract

Primary biliary cholangitis (PBC) is a chronic inflammatory autoimmune liver disease characterized by inflammation and damage of small bile ducts. The NLRP3 inflammasome is a multimeric complex of proteins that after activation with various stimuli initiates an inflammatory process. Increasing data obtained from animal studies implicate the role of NLRP3 inflammasome in the pathogenesis of various diseases. Galectin-3 is a β-galactoside-binding lectin that plays important roles in various biological processes including cell proliferation, differentiation, transformation and apoptosis, pre-mRNA splicing, inflammation, fibrosis and host defense. The multilineage immune response at various stages of PBC development includes the involvement of Gal-3 in the pathogenesis of this disease. The role of Galectin-3 in the specific binding to NLRP3, and inflammasome activation in models of primary biliary cholangitis has been recently described. This review provides a brief pathogenesis of PBC and discusses the current knowledge about the role of Gal-3 in NLRP3 activation and PBC development.

## 1. Primary Biliary Cholangitis

Primary biliary cholangitis (PBC) is a chronic inflammatory autoimmune liver disease characterized by destructive lymphocytic inflammation of intrahepatic small bile ducts, increased serum levels of anti-mitochondrial antibodies (AMA) specific for mitochondrial autoantigens and higher incidence in the female population [1,2]. The key serological finding in PBC is the presence of disease-specific AMA antibodies that can be detected in more than 95% of patients [3]. These autoantibodies are specific to the antigenic determinant E2, located within different subunits of the complex of dehydrogenase enzyme, located in the mitochondrial membrane. These subunits are: 2-oxo-acid (2OADC-E2), pyruvate (PDC-E2), branched-chain 2-oxo-acid (BCOADC-E2) and 2-oxo-glutarate (OGDC-E2) [1]. The immunodominant autoantigen in PBC is PDC-E2. Loss of tolerance to PDC-E2 is accompanied by the development of cholangiocytes damage [1]. Until 2015, the name for this disease in the official nomenclature was primary biliary cirrhosis (PBC), but the abbreviation remained the same [4].

The mechanisms of the disease onset and the pathogenesis are very complex and imply a loss of tolerance to autoantigens present in cholangiocytes, leading to the inflammation development with consequent damage of bile ducts, the development of cholestasis and liver fibrosis. There are several factors that play a role in PBC development: exposure to certain substances present in the environment, immunogenic predisposition, epigenetic control of the biliary epithelium, congenital and acquired immune response and disorder of bile acid production. Only recently, the combined role of immune mechanisms, disorders of bile salt production, biliary transport function and cholangiocyte apoptosis has been considered in the pathogenesis of PBC [5].

Now, it is considered that, in PBC, combined genetic, epigenetic and environmental factors are necessary for the initial damage of the biliary epithelium, followed by the immune/inflammatory response to damaged cholangiocytes that is responsible for the chronicity of the disease [6].

Although the pathogenesis and etiology of PBC is still unclear, the association of liver fibrosis in PBC and cancerogenesis is quite clear. PBC has been described as the risk factor for hepatocellular carcinoma [7,8], but a case of cholangiocellular carcinoma developed in the PBC patient has not been described and it is in accordance with the previous report that the liver microenvironment in biliary tract cancers is immunosuppressive [9]. Some cases of combined hepatocellular and cholangio cellular carcinoma have been reported [10,11].

## 2. Genetic and Environmental Factors in PBC

The results of numerous studies indicate the role of genetic factors as a risk factor in PBC development, showing a concordance of 63% in monozygotic twins and a higher incidence rate in certain families with a relative risk of 9.13–10.5 in the relatives in the first line of the relationship to 1.66 in the fifth line of the relationship [12,13]. The association of certain gene variants of HLA, but also non-HLA genes, and a higher risk for PBC development has been described. Products of the genes associated with increased PBC risk play the role in the modulation of PBC pathogenesis, but are also influenced by environmental factors [14]. The strongest association between PBC and *non*-HLA genes has been indicated for: *IL12RB2, STAT4, STAT1, CD80, IL12A, NFKB1, IL7R, TNFSF15, CXCR5, DDX6* and *RF8* genes whose products control the several immune reactions, e.g., antigen presentation, lymphocyte differentiation and immune response to microbes [15,16,17]. It seems that among these genes the most important role in the PBC pathogenesis play the genes whose products control IL-12 signaling and thus activation and differentiation of naive T lymphocytes toward inflammatory Th1 cells, but also by stimulation of IFN-γ production inhibit the Th17 cells development, thus playing bidirectional roles in PBC development [18]. Immunohistochemical studies of the livers obtained by PBC patients indicate the significance of the IL-12 and IL-23 signaling in PBC [19]. Some alleles of *CCL20* are associated with lower PBC risk [12], since CCL20:CCR6 interaction plays the role in differentiation and function of the mucosal lymph tissue, Th17 cells homing, biliary epithelium damage and function of effector CD8+ T cells in portal tracts [20,21,22].

It is considered that several environmental factors could be the triggers for the loss of tolerance to mitochondrial antigens, unleashing thus the key initial step in PBC development. Recurrent urinary tract infections caused by *Escherichia coli* induce the production of specific anti-PDC-E2 antibodies and thus increase the risk for PBC development [23]. It is proposed that similar mechanisms explain the increased risk of PBC developing after infections with other microorganisms such as: *Novosphingobium aromaticivorans*, *Helicobacter pylori*; *Chlamydia pneumoniae*; *Mycobacterium gordonae*; *Epstein-Barr* virus; *Cytomegalovirus* and *Toxoplasma gondii* [24,25]. In animal models of the disease it has been shown that xenobiotics, such as 2-octinoic acid, play a role in the pathogenesis of PBC [26].

## 3. Immune Dysregulation in PBC

PBC is characterized by multilineage immune dysregulation and a loss of auto-tolerance, resulting in targeted cholangiocyte damage [27,28]. Disease-specific anti-mitochondrial antibodies bind to immunodominant epitopes of PDC-E2 located in the inner mitochondrial membrane. PDC-E2 contains a lipoic acid-lysine bond necessary for this recognition and activation of the immune system [29,30]. Regardless of the fact that this autoantigen is ubiquitous, the targeted damage of cholangiocytes is probably a consequence of aberrant modification of mitochondrial PDC-E2, keeping intact the immunodominant epitope, within the apoptotic bodies of biliary epithelial cells. This immunogenic complex is recognized by circulating antibodies resulting in the formation of antigen–antibody complexes [31,32]. Increased levels of AMA in the serum and infiltration of the liver and portal spaces of PBC patients with CD4+ T and CD8+ T lymphocytes indicate the role of a specific immune response in PBC pathogenesis [33,34]. The population of effector memory CD8+ T lymphocytes, localized around the portal tracts in the livers of PBC patients recognizes antigenic sequences within the PDC-E2 domain that contain lipoic acid and contributes to targeted damage to the biliary tract [35,36]. Th17, Th1 and follicular helper T cells contribute to the development of the disease [37,38]. The stage of advanced fibrosis is associated with a shift of the immune response to the Th17 phenotype, with dominant production of IL-17, IL-6 and TGF-β, which has been confirmed in the infiltrates of liver sections obtained from PBC patients [39,40]. Follicular T helper cells, also found in greater number in the livers of patients with PBC, provide the necessary help to B lymphocytes to differentiate to cells capable of the production of an altered isotype of the specific antibodies [36]. A decreased number of Treg cells has been found in the livers of patients with PBC [41,42]. 

The importance of innate immunity in the development of PBC is indicated by the presence of granulomas and polyclonal IgM, but the mechanisms of innate immunity alone, without contribution of the acquired immunity, are not sufficient to cause a break in autotolerance [43]. Cholangiocytes express Toll like receptors (TLRs), which activated by various ligands including products of microorganisms, produce the proinflammatory mediators such as NF-κB, CX3CL1 and IL-8 that contribute to biliary epithelial cell damage and recruitment of immune effector cells into the portal tracts [44,45]. Increased expression of CX3CL1 in damaged cholangiocytes attracts CD4+ T and CD8+ T lymphocytes, which are found to be more abundant in the liver of patients with PBC [44]. A recent study indicated an increase in the number of suppressor cells of myeloid origin in the liver of patients with primary biliary cholangitis and correlation of their number with the biochemical parameters of the disease: the concentration of ALP and serum bilirubin [46]. In the presence of circulating AMA and apoptotic cholangiocytes, the expression of the proinflammatory cytokine IL-12 is increased in the macrophages, which indicates the link between apoptosis of the cholangiocyte and the innate immune system response [47]. A higher prevalence of NKT cells has been shown in the liver of PBC patients compared to healthy controls, as well as NK cells, that also contribute to cholangiocyte damage, autoantigen release and activation of autoreactive T lymphocytes [48,49]. Mucosal associated invariant T cells (MAIT cells), invariant mucosal T cells, are a relatively novel population of innate immune cells that produce inflammatory cytokines IFN-γ, TNF-α and IL-17, or independently, or by stimulation with microorganisms, present in a smaller percentage in the liver and blood of patients with PBC compared to controls [1,49,50].

All metabolites, nutrients and bacterial products as well as cells of the immune system found in the intestine through the portal circulation firstly go to the liver [51,52]. In addition, through the enterohepatic circulation of primary and secondary bile acids and immunoglobulins, the liver directly affects homeostatic processes and absorption in the intestine [53,54,55]. Intestinal microbiome dysbiosis is either the result, or response to the development of certain diseases and may affect nutrient degradation, damage to epithelial tight junctions and thus increase the intestinal permeability [56]. Altered function of the intestinal epithelial barrier can result in increased diffusion of pathogen-associated molecular patterns of microorganisms (PAMP), damage-associated molecular patterns (DAMPs), free fatty acids and endotoxin into portal circulation all the way to the hepatic sinusoids and thus can be a trigger for liver damage and dysregulation of immune reactions in the liver, and if there are other factors that can contribute to the maintenance of the damage, chronic liver disease develops [57]. Kupffer cells are the first line of defense against pathogens that have reached the liver from the intestine [58]. If Kupffer cells activated by bacteria, lipopolysaccharide or toxins that have reached the liver obtain M1 phenotype, they produce proinflammatory cytokines IL-6, TNF and IL-1β, which activate profibrotic stellate cells in the liver, while M2 phenotype of Kupffer cells has the tolerogenic function, since these cells produce IL-10 and TGF-β, which increases the activity of immunosuppressive Treg cells [59]. Thus, microorganisms that reach the liver may be responsible for the development of PBC, because they play a role of innate immune responses triggers, which result in dysregulation of the immune system in the liver.

Intrahepatic MAIT cells are T cells with invariant TCRs that play the key role in the immune response to antigens raised by vitamin B metabolism mediated by intestinal bacteria. These antigens are presented to MAIT cells in the liver within MHC I molecules by macrophages, cholangiocytes, and B cells [59,60]. MAIT cells, also called the biliary epithelial defense system, are located mainly in the portal tracts where they can be activated by presented antigens and initiate a localized immune response that aims to control pathogenic microorganisms that reach the liver, including the recruitment of effector lymphocytes in the liver [61]. The number of MAIT cells in the liver of patients with PBC is significantly reduced compared to healthy controls, in contrast to conventional CD4+ T and CD8+ T lymphocytes, whose presence in the liver of PBC patients is significantly increased [60]. During the therapeutic treatment with ursodeoxycholic acid, the number of MAIT cells in the liver does not normalize, even if disease improvement is registered, which could be one of the mechanisms that explain progressive liver damage regardless of the therapeutic response [62].

Secretory IgA produced by plasma cells present in the portal system and secreted into the intestinal lumen together with bile acids may also play a role in protecting the biliary epithelium from microorganisms. Within the intestinal tract, secretory IgA binds directly to bacteria and thus traps them inside the mucus, allowing them to be expelled from the intestine by feces. In addition, these antibodies neutralize bacterial toxins and interfere with the binding of bacteria to the apical surfaces of enterocytes [63]. Lower concentrations of IgA on the intestinal surface of enterocytes of the duodenum in patients with PBC compared to healthy controls were observed, and it contributes to the disruption of the epithelial barrier [54,64].

Dysbiosis results in a change in immune activity in the intestine, increases the polarization of CD4+ T lymphocytes to the Th17 phenotype and increases the production of proinflammatory cytokines crucial for host defense against pathogens. Infection of mice with the bacterium *Citrobacter rodentium* results in apoptosis of enterocytes that release autoantigens and stimulate the differentiation and proliferation of autoreactive T lymphocytes [60]. Altered fecal microflora is present in patients with PBC [65,66]. These observations indicate the important role of microorganisms in the initiation of pathological processes in PBC. Experimental models of PBC induced by infection with different microorganisms also indicate an important role of stimulation of innate immunity in triggering the pathological process. Infection of NOD.B6-Idd10/Idd18 mice with *Escherichia coli* results in an increase of anti-AMA titers and development of a severe form of cholangitis [23,24]. Additionally, NOD, C57BL/6 and SJL mice infected with bacterium *Novosphingobium aromaticivorans* have increased levels of specific antibodies in the sera and activated T cells that induce bile ducts damage [67]. *Novosphingobium aromaticivorans* contains molecules homologous to PDC-E2 autoantigen, which in infected mice initiate the production of PDC-E2 specific IgG and liver damage almost identical to those lesions developed during PBC in humans [68], with an increased number of NKT cells in the liver, with increased expression of the CD1d molecule [67]. This disease can be transferred by CD4+ and CD8+ T lymphocytes from a diseased mouse to healthy mice.

## 4. NLRP3 Inflammasome

One of the mechanisms of innate immunity activation and induction of an inflammatory response includes involvement of inflammasomes. Inflammasome is a multimeric protein complex present in the cytoplasm that after oligomerization activates caspase-1 leading to the release of mature pro-inflammatory cytokines IL-1β and IL-18, but also to the inflammatory cell death, pyroptosis [69]. The most studied and the best known inflammasome is NLRP3. The NLRP3 inflammasome consists of a sensor (NLRP3), an adaptor molecular apoptosis-associated speck-like protein containing a caspase-recruitment domain (ASC) and a precursor of effector molecule caspase 1, pro-caspase-1 [70,71]. It seems that the limiting factor for the activation of the NLRP3 inflammasome is the expression of NLRP3 itself [72]. The best described model of NLRP3 inflammasome activation is the canonical two-step model of the activation that demands two independent and coincident steps—transcription (priming) and oligomerization (activation) [73]. The priming signal that licenses the cell upregulates the expression of NLRP3 and pro-IL-1β, while a coincident second signal has the main role in the assembly of subunits and forming of the functional inflammasome [74,75]. The first signal that induces expression of NLRP3 and pro-IL-1β requires the activation of nuclear factor kappa B (NF-κB), which is mostly activated by the stimulation of tumor necrosis factor (TNF) receptor, cytokine receptors or Toll like and NOD2 receptors by PAMPs or DAMPs. Activated NF-κB binds to pro-IL-1β and NLRP3 promoters and thus induced synthesis of these molecules [72]. Additionally, the first signal involves a post-translational regulation of inflammasome components, including deubiquitination of NLRP3, phosphorylation and ubiquitination of ASC [76]. After the priming proceeds the second phase, the oligomerization of the NLRP3 with an ASC, which induced activation of pro-caspase-1 and release of active forms of inflammatory cytokines IL-18 and IL-1β [77,78]. The second signal for NLRP3 inflammasome activation is mediated by various structurally unrelated agonists, including environmental crystalline pollutants like silica, asbestos, crystalline monosodium urate, extracellular osmolarity, β-amyloid protein and α-synuclein accumulation, and pathogen-derived ligands, such as pore-forming toxins RNA–DNA hybrids, and numerous bacterial, fungal, protozoan and viral pathogens, which induce K+ efflux and a large pore membrane formation [75]. Increased membrane permeability allows influx of PAMPs or DAMPs into the cell, which activate the NLRP3 inflammasome. Secondary stress-related signals also include reactive oxygen species derived from mitochondria and lysosomal rupture signals that promote NLRP3 inflammasome complex assembly and activation [78,79]. Results from animal model studies indicate that oxidized low-density lipoprotein and cholesterol crystals [80,81], uric acid accumulation and monosodium urate crystals [82,83] also trigger NLRP3 inflammasome activation. Beside the canonical mode of NLRP3 inflammasome activation, there are several non-canonical activation pathways that depend on the activity of caspase-5 and caspase-4 in humans and on caspase-11 in mice [84] and caspase-8 [85,86,87,88,89]. It has been shown that there is no caspase-8 activation and releasing of mature form of IL-1β in bone marrow-derived dendritic cells lacking expression NLRP3 and ASC genes [88,89].

The NLRP3 inflammasome is expressed in various immune cells and is mainly well characterized in macrophages, but also in neutrophils, monocytes and dendritic cells [69,90]. Besides, the NLRP3 inflammasome is expressed in an extensive range of non-immune cells, including cholangiocytes where its activation leads to IL-18 release but not IL-1β [91].

## 5. Galectin-3

Galectins are carbohydrate-binding proteins, involved in a number of physiological processes, such as inflammatory and immune response, cell migration, autophagy and cell signaling. Galectins are defined as a family of proteins that within characteristic carbohydrate-recognizing domains contain a conserved β-galactoside binding site containing about 130 amino acids (carbohydrate recognition domain, CRD) that binds β-galactoside [92]. Galectins are synthesized in the cytosol, spend most of their life in the cytosol or in the nucleus, and bind galactoside ligands only after nonclassical secretion that bypasses the Golgi complex [93]. Galectins also interact with various galactose-free molecules, even within CRDs there are specific sites that bind to other types of macromolecules [94,95]. Galectin-3 (Gal-3) is a chimeric galectin containing two domains, one recognizing carbohydrates and the other long, flexible domain at the N-terminus [96,97]. The N-terminal domain contains collagen-α-like repeating sequences rich in glycine, proline and tyrosine [98,99]. N-terminus of Gal-3 mediates multivalent associations involving N-terminus interactions with the N-terminus of another molecule and N-terminus interactions with CRD [100]. The N-terminus in the presence of different ligands (multivalent carbohydrates) allows polymerization and the formation of a pentameric form of galectin-3 that cross-links glycans on the cell surface thus activating intracellular signaling pathways, and affecting the expression of various genes and altering cell function [101,102,103,104].

### 5.1. Functions of Gal-3

Galectin-3 is a ubiquitous molecule, presents both extra- and intra-cellularly but also as a membrane molecule. It is mostly expressed in epithelial, endothelial and cells of the immune system [105,106]. Within the cells galectin-3 is present in the nucleus, mitochondria or cytosol, and its distribution is influenced by the type of the cell and the phase of the cell cycle [107,108]. Galectins interact with a variety of cytosolic and nuclear ligands, which are structurally proteins, and thus regulate signaling pathways [109]. Galectin-3 interacts with small GTPases HRAS [110] and KRAS, activates kinases of Raf-1/MEK/ERK signaling pathway and induces proliferation of the cells [111]. Since Gal-3 interacts with basal bodies and centrosomes, in the absence of galectin-3 the organization of microtubules and morphogenesis of primary cilia [112] and respiratory epithelial cilia motility [113] are disturbed. Gal-3 also associates with the NuMa protein (nuclear apparatus mitotic protein), the key regulator of mitosis [114]. Gal-3 also interacts with the ALG-2 component of the endosomal sorting complexes required for transport, which regulates the transport of endosomes and participates in the remodeling of the cell membrane [115]. This interaction reduced the expression of TCR on T lymphocyte membranes, the intracellular trafficking of epidermal growth factor receptors [116] and the release of human immunodeficiency virus-1 from the cell [117]. Cytosolic galectin-3 releases β-catenin from the Wnt complex allows its translocation into the nucleus and thus affects Wnt signaling [118]. Gal-3 interacts with the Bcl-2 molecule and thus performs antiapoptotic effects in the cell [119]. The NWGR motif of galectin-3, which is also part of the CRD domain, binds to the Bcl-2 molecule [120].

Galectin-3 constantly migrates between the cytoplasm and the nucleus. In the nucleus, galectin-3 participates in pre-mRNA splicing, in the regulation of gene expression and associates with small nuclear ribonucleoproteins [121,122]. In addition, Gal-3 allows the stable binding of CREB and Sp1 transcription factors to the promoter regions of the gene responsible for cyclin D1 synthesis [123].

Galectin-3 rapidly surrounds damaged endocytic vesicles because upon vesicle disruption, due to the rapid exposure to the cytosol of glycans present in the vesicle lumen. This phenomenon was firstly observed when the binding of galectin-3 to vacuoles containing *Shigella* was described [124]. Later the binding of galectin-3 to endosomes containing adenoviruses was also described [125]. Galectins also accumulate around endosomes destroyed by protein aggregation [126,127]. These facts may indicate that galectins use exocytosis and the process of autophagy for their secretion into the extracellular space [128].

Galectins from the extracellular space can re-enter cells by endocytosis [129] and then retain in endocytic vesicles [130] and endosomes [131]. Extracellular galectins, among other things, modify interactions with microorganisms [132,133,134]. It has recently been shown that galectins can bind to glycans on the surface of helminths and thus enable the effector functions of innate immunity [135]. When it comes to the action of galectin-3 at the level of the organism, the functions of galectins in the extracellular space, on the cell membrane and intracellularly, are generally described separately [134,136]. However, galectin-3 from the extracellular space endocytose very rapidly and move rapidly between endo- and exocytic vesicles [131]. Establishing optimal levels of galectin-3 in the cell and extracellular space affects the movement of galectin through vesicles and thus directly affects the cellular processes that this molecule controls.

### 5.2. The Role and Importance of Galectin-3 in the Regulation of the Immune Response

Constitutively, Gal-3 is expressed in monocyte/macrophage cell lines, as well as in dendritic cells, polymorphonuclear cells and mastocytes, while T and B lymphocytes synthesize Gal-3 only after activation [137]. 

Galectin-3 participates in the differentiation of macrophages, B lymphocytes and dendritic cells [138,139,140]. Expression of Galectin-3 is increased in human monocytes during in vitro differentiation into macrophages [138]. Galectin-3 is important for the adhesion and chemotaxis of monocyte/macrophage cells and the migration of these cells through the endothelium [101], and due to its function as receptor for PAMPs enhances the phagocytic and microbicidal activity of phagocytes. Specifically, this molecule recognizes and binds to glycoconjugates, on the surface of pathogens such as: *Neisseria gonorrhoeae, Leishmania major, Schistosoma mansoni* and *Trypanosoma cruzi*, and also stimulates opsonization [141,142,143,144,145].

### 5.3. Galectin-3 and Liver Diseases

Galectin-3 plays very important roles in the pathogenesis of inflammatory liver diseases and liver malignancies [146,147]. Several previous studies have confirmed altered galectin-3 expression in the liver and serum of patients with hepatocellular carcinoma, steatohepatitis and cirrhosis [146]. Galectin-3 expression can be used as a prognostic factor in hepatocellular carcinomas [147], since higher expression of galectin-3 in nuclei of cancer cells corresponds to a poorer prognosis [146]. Suppressing the expression of galectin-3 in the nucleus of tumor cells facilitates apoptosis and increases the sensitivity of cholangiocarcinoma cells to chemotherapeutic agents [147]. A similar effect of galectin-3 was shown in animal model of PBC induced by immunization with xenobiotic mixed with a strong adjuvant [148]. Galectin-3 exerts the antiapoptotic effect in cholangiocytes, and thus most likely reduces the further release of autoantigens leading to the attenuation of the autoimmune process [148].

### 5.4. Galectin-3, Inflammasome and PBC

Activated inflammasome in the liver macrophages has a significant role in the onset and development of liver diseases [149]. Significant hepatocyte pyroptosis, inflammation and fibrosis have been shown in the livers of transgenic mice that constitutively express active NLRP3 [150]. The importance of galectin-3 in the activation of inflammasome and the consequent development of primary biliary cholangitis has been shown in two different animal models of the disease. 

In a model of spontaneously developing PBC in dnTGF-βRII mice, galectin-3 has been shown to directly stimulate inflammasome activation and consequent development of the Th17 immune response resulting in manifestations of autoimmune cholangitis and the development of fibrosis [151]. It is known that NLRP3 can be activated by DAMPs [152], and it also known that Gal3 is considered as a DAMP molecule [153]. It has been shown that the activation of NLRP3 in hepatic macrophages stimulated with deoxycholic acid was Gal-3 dependent [151]. They also showed by immunoprecipitation and a biolayer interferometry assay, a direct binding of N-terminal domain of Gal-3 to NLRP3, which resulted in the activation of the inflammasome. Significantly reduced inflammasome activation has been shown in Gal3^−/−^ cells. Recombinant Gal-3 increased NLRP3 activation in Gal-3^−/−^ cells, suggesting that extracellular Gal-3 also could contribute to inflammasome activation. “Lattice” formation by extracellular Gal-3 also could mediate crosslinking of the molecules [154], leading to stronger inflammasome activation.

We used the model of PBC induced by *Novosphingobium aromaticivorans* infection of C57BL/6 mice to further explore the role of Gal-3 in PBC pathogenesis and its role in NLRP3 inflammasome activation. We found significantly higher percentage of NLRP3 expressing dendritic cells and macrophages, higher production of IL-1β and higher expression of NLRP3 and ASC in the livers of Gal-3^+/+^ mice early after infection with *N. aromaticivorans* in comparison with the group of Gal-3^−/−^ mice [155].

Further, the in vitro stimulation of dendritic cells by bacterium *N. aromaticivorans* significantly increases the expression of NLRP3 only in cells isolated from galectin-3 positive mice [155], while the in vitro stimulation of peritoneal macrophages isolated from Gal-3 positive mice by this bacterium results in increased protein level of NLRP3 inflammasome, increased production of IL-1β and increased activity of caspase-1 [155]. These results are consistent with the results of a previous study where it has been shown that deletion of *Lgals3* in dnTGF-βRII mice results in poorer activation of the inflammasome, attenuation of the Th17 immune response and significant improvement of bile duct inflammation [151].

**Figure 1:**
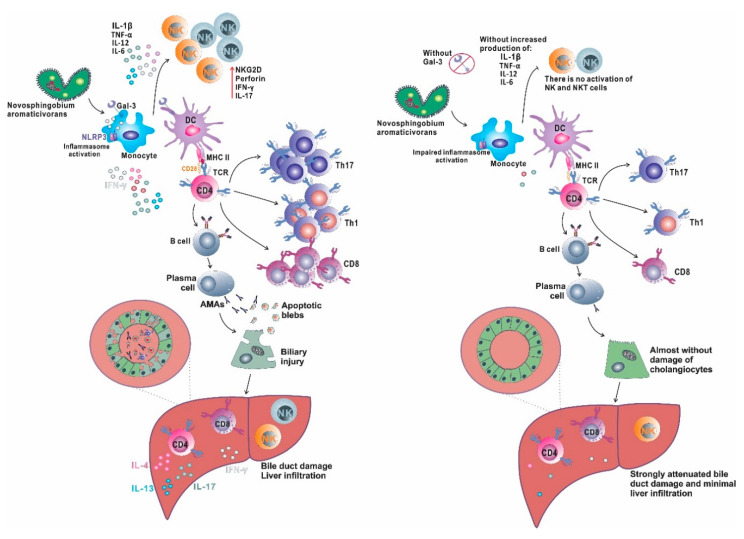
Gal-3 in inflammasome activation and PBC development. *Novosphingobium aromaticivorans* infection activates innate immune cells (monocytes/macrophages) and in the presence of Gal-3 (left side) induces NLRP3 inflammasome activation. Activated macrophages produce a large amount of proinflammatory cytokines that activates NK cells and induce adequate activation of dendritic cells that are capable to activate autoreactive T lymphocytes (probably by mechanism of molecular mimicry) and induce their differentiation toward inflammatory Th1 and Th17 phenotype, and stimulate B cells to produce autoantibodies, AMA. Apoptosis of cholangiocytes induced by inflammatory process results in PDC-E2 antigen exposure in apoptotic blebs, which is recognized by produced AMA and results in antigen–antibody complex formation that contributes to cellular injury. Bile duct damage is the result of intrahepatic accumulation of inflammatory Th1 and Th17 cells, inflammatory and cytotoxic CD8 cells, activated, IL-17 producing NK and NKT cells that promote biliary injury, accompanied by autoantigen release that leads to perpetuation of autoimmune process. In the absence of Gal-3 (right side) there is no sufficient inflammasome activation followed by inadequate activation of dendritic cells and later insufficient activation of T and B lymphocytes. There is insignificant liver infiltration with inflammatory T, NK and NKT cells insufficient to trigger autoimmune process, and there is no biliary damage.

Inflammasome activation plays an important role in the pathogenesis of liver diseases (metabolic and inflammatory) that are triggered by weak but repetitive stimuli. On the other hand, strong stimulators of the immune system used in the induction of autoimmune hepatitis activate primarily innate immunity receptors [156]. *N. aromaticivorans* is bacteria with an atypical cell wall containing glycosphingolipids, molecules similar to molecules in the eukaryotic membrane, which generally do not cause inflammation and tissue damage, although it is detected in the mucosa of the digestive tract [157]. Therefore, it can be assumed that the activation of innate immunity by the bacterium and the inflammatory response to *N. aromaticivorans* in mice are triggered by inflammasome activation, namely, by the integration of individually insufficient signals, which creates conditions for subsequent activation of immune cells and cholangitis. The almost complete absence of disease in *Lgals3*^−/−^ mice and significantly attenuated disease in the wild type receiving galectin-3 inhibitor are consistent with previous data on the role of galectin-3 in inflammasome activation [151,158]. Probably the dominant role in the activation of dendritic cells during PBC induction with *N. aromaticivorans* plays inflammasome. Our results are summarized in Figure 1 and indicate that Gal-3 deficient mice do not develop PBC after *N. aromaticivorans* infection due to insufficient NLRP3 inflammasome activation and subsequent dendritic cells activation, which results in inadequate activation and differentiation of other immune cells that play a significant role in PBC pathogenesis, particularly NK, NKT and IL-17 producing T cells.

## 6. Conclusions

Gal-3 (Table 1) and the inflammasome (Table 2) play the important role in different liver diseases including PBC. Gal-3 plays an inflammatory role in experimental models of PBC by direct interaction with NLRP3 and stimulation of inflammasome activation in liver macrophages. Activated inflammasome in liver macrophages induce production of proinflammatory cytokines that affect integrity of biliary epithelial cells and triggers their damage. Besides immune cells, NLRP3 component of inflammasome is expressed in biliary epithelial cells in primary sclerosing cholangitis, and its lower expression during time and insufficient signaling may be associated with the development of cholangiocarcinoma [159]. Increased expression of Gal-3 in the preneoplastic and early neoplastic stages of intrahepatic cholangiocarcinoma has been reported [160], while intranuclear Gal-3 expression in distal cholangiocarcinoma is marked as a negative prognostic factor [161]. Gal-3 expression in biliary epithelial cells is increased during PBC, and since these cells play an active role in PBC pathogenesis, the role of Gal-3 in NLRP3 inflammasome activation in cholangiocytes in PBC development needs to be elucidated, but also the role of this axis in the development of intrahepatic cholangiocarcinoma. It is clear that inhibition of galectin-3 signaling and inflammasome activation may be a potential target for the treatment of primary biliary cholangitis, however further studies are needed to explore the balance between beneficial and harmful effects of Gal-3 in different phases of PBC pathogenesis and different types of cells that play a roles in PBC pathogenesis.

## Figures and Tables

**Table 1:** Immunoregulatory role of galectin-3 in inflammatory and malignant liver diseases.

Disease	Model	Role of Gal-3	Mechanisms	References
Experimental Primary Biliary Cholangitis (PBC)	Xenobiotic induced PBC in Gal-3 deficient mice	Protective	Gal-3 protects biliary epithelial cells from apoptosis and thus attenuates autoantigen release	[148]
	*Novosphingobium aromaticivorans* induced PBC in Gal-3 deficient mice	Proinflammatory	Gal-3 is necessary for the activation of NLRP3 inflammasome in liver macrophages and subsequent IL-17 mediated inflammatory process	[155]
	Spontaneously developed autoimmune cholangitis in dnTGF-βRII mice and Gal-3 deficient mice	Proinflammatory	Gal-3 is an initiator of inflammatory signaling and mediation of the activation of NLRP3 inflammasome and IL-17 proinflammatory cascades	[151]
Experimental Hepatitis	MCMV induced hepatitis Gal-3 deficient mice	Antiinflammatory, protective	Gal-3 attenuates TNF-α-mediated death of hepatocytes	[162]
	α-galactosylceramide induced hepatitis in Gal-3 deficient mice	Proinflammatory	Gal-3 regulates the capacity of DCs to support NKT-cell-mediated liver injury	[163]
	Con A-induced hepatitis in Gal-3 deficient mice and Gal-3 inhibitor treated mice	Proinflammatory	Gal-3 promotes the influx of mononuclear cells and proinflammatory CD4^+^ cells in the liver and decreases influx of IL-10 expressing CD4^+^ T cells and F4/80^+^ macrophages	[164]
Experimental Non-Alcoholic Fatty Liver Disease	High-fat diet nonalcoholic steatohepatitis induced in Gal-3 deficient mice	Proinflammatory	Gal-3 increases hepatic accumulation of advanced lipoxidation endproducts, up-regulates lipid synthesis and oxidation causing more fat deposition, oxidative stress, and inflammation	[165]
	High-fat diet induced steatohepatitis and liver steatosis in Gal-3 deficient mice	Proinflammatory	Increased mRNA expression of CCL2, F4/80, CD11c, TLR4, CD14, NLRP3 inflammasome, IL-1β, IL-13 and IL-33 in the liver	[166]
Experimental Hepatocellular Carcinoma	N-diethylnitrosamine induced hepatocellular carcinoma in Gal-3 deficient mice	Pro-tumor effects	Gal-3 promotes motility and invasion of hepatoma cells by an autocrine pathway	[167]

**Table 2:** Immunoregulatory role of inflammasome in inflammatory and malignant liver diseases.

Disease	Model	Role of Inflammasome	Mechanisms	References
Experimental Hepatitis	α-galactosylceramide induced hepatitis in NALP3 deficient mice	Proinflammatory	NALP3 promotes expression of proinflammatory cytokines (IL-6, and TNF-α)	[168]
	Con A-induced hepatitis in Nlrp3 deficient mice	Proinflammatory	NLRP3 inflammasome dependent IL-1β production is crucial for hepatitis development	[169]
Experimental Hepatocellular Carcinoma	N-diethylnitrosamine induced hepatocellular carcinoma in AIM2 deficient mice	Pro-tumor effects	AIM2 inflammasome component promotes inflammation during carcinogenic liver injury and contributes to genotoxic HCC development in mice	[170]
Experimental Non-Alcoholic Fatty Liver Disease	Western-lifestyle diet with fructose in drinking water induced Non-Alcoholic Fatty Liver Disease in Nlrp3 deficient mice	Antiinflammatory	NLRP3 maintains normal gut immune response and attenuates adipose tissue inflammation	[171]
Experimental Non-Alcoholic Fatty Liver Disease	Methionine-choline-deficient diet induced Non-Alcoholic Fatty Liver Disease in Nlrp3 deficient mice	Proinflammatory	NLRP3 stimulates IL-1β and IL-18 secretion induced by palmitic acid stimulation and promotes liver inflammation	[172]
Experimental Non-Alcoholic Fatty Liver Disease	Short-term choline-deficient amino acid-defined diet, to induce isolated hepatic steatosis or long-term exposure, to induce severe steatohepatitis and fibrosis in Nlrp3 deficient mice	Proinflammatory and profibrotic	NLRP3 stimulates IL-1β secretion and promotes liver inflammation and fibrosis	[173]

## Abbreviations

2OADC-E2	2-oxo-acid E2 dehydrogenase
AMA	anti-mitochondrial antibodies
ASC	apoptosis-associated speck-like protein
BCOADC-E2	branched-chain 2-oxo-acid E2 dehydrogenase
CRD	carbohydrate recognition domain
DAMPs	damage-associated molecular patterns
Gal-3	galectin-3
MAIT cells	mucosal associated invariant T cells
NF-κB	nuclear factor kappa B
OGDC-E2	2-oxo-glutarate E2 dehydrogenase
PBC	primary biliary cholangitis
PDC-E2	pyruvate E2 dehydrogenase
PAMPs	pathogen-associated molecular patterns of microorganisms
TLRs	toll like receptors
TNF	tumor necrosis factor
